# Supplementary material for: Effect of Sheep’s Whey Edible Coatings with a Bioprotective Culture, Kombucha Tea or Oregano Essential Oil on Cheese Characteristics
Source: Foods. 2024 Dec 20;13(24):4132. doi: 10.3390/foods13244132 (PMC11675235; doi:10.3390/foods13244132)
Supplement: Supplementary file 1 [file foods-13-04132-s001.zip › foods-3346068-supplementary.pdf]

SUPPLEMENTARY MATERIALS

Table S1. Two-way Anova of the physicochemical characteristics of the samples. MDC = moisture in defatted cheese; FDM = fat in dry matter.

		F	*p*
	Product	20.4	0.000
Dry matter	Time	48.9	0.000
	Interaction	4.2	0.000
	Product	11.3	0.000
MDC	Time	406.1	0.000
	Interaction	6.4	0.000
	Product	7.1	0.000
FDM	Time	1051.9	0.000
	Interaction	19.8	0.000
	Product	1.0	0.042
pH	Time	13.0	0.000
	Interaction	4.0	0.000
	Product	7.0	0.000
Titratable acidity	Time	1052.0	0.000
	Interaction	20.0	0.000
	Product	19.0	0.000
a_w	Time	28.0	0.000
	Interaction	5.0	0.000

Table S2. Two-way Anova of the textural parameters of the samples.

		F	*p*
	Product	31.7	0.000
Hardness	Time	1046.9	0.000
	Interaction	10.2	0.000
	Product	6.1	0.000
Adhesiveness	Time	24.9	0.000
	Interaction	1.8	0.048
	Product	8.6	0.000
Chewiness	Time	149.1	0.000
	Interaction	8.9	0.000
	Product	4.1	0.002
Cohesiveness	Time	27.4	0.000
	Interaction	1.7	0.059

Table S3. Two-way Anova of the color parameters of the rind and of the paste of cheese samples.

		RIND		*PASTE*	
		F	*p*	*F*	*p*
	Product	9.2	0.000	1.5	0.195
L*	Time	276.4	0.000	86.8	0.000
	Interaction	4.2	0.000	1.5	0.127
	Product	8.7	0.000	0.3	0.907
a*	Time	66.5	0.000	16.7	0.000
	Interaction	3.2	0.000	0.6	0.906
	Product	11.1	0.000	0.4	0.833
b*	Time	149.7	0.000	126.2	0.000
	Interaction	2.3	0.007	1.4	0.156

Table S4. Two-way anova of the microbial counts of the samples.

		F	*p*
	Product	3.9	0.005
Lactobacilli sp.	Time	1.0	0.371
	Interaction	3.1	0.003
	Product	2.0	0.097
Lactococci sp.	Time	0.8	0.457
	Interaction	3.8	0.000
	Product	11.5	0.000
Yeasts and molds	Time	67.4	0.000
	Interaction	5.5	0.000
